# The association between trait anxiety and the fear of being laughed at in college: A preliminary study with a Brazilian sample

**DOI:** 10.1002/brb3.2757

**Published:** 2022-09-30

**Authors:** Tainá S. Rêgo, Diógenes E. S. Pires, Eline M. Melo, Juan‐Pablo Aristizabal, Antonio Pereira

**Affiliations:** ^1^ Laboratory of Neuroprocessing, Institute of Technology Federal University of Pará Belém Pará Brazil; ^2^ Graduate Program in Electrical Engineering Federal University of Pará Belém Pará Brazil; ^3^ Graduate Program in Cell Biology and Neuroscience Federal University of Pará Belém Pará Brazil; ^4^ Graduate Program in Neuroscience and Behavior Federal University of Pará Belém Pará Brazil

**Keywords:** anxiety, bullying, college students, gelotophobia, mental health

## Abstract

**Aim:**

To evaluate how gelotophobia correlates with trait anxiety in a sample of Brazilian college students.

**Methods:**

We evaluated the association of GELOPH < 15 > scores with both self‐reported experiences of bullying victimization and trait anxiety measures assessed by the State‐Trait Anxiety Inventory (STAI). The study consisted of a sample of 65 adult volunteers (*M* = 21.48, SD = 2.54 years, 38 females), recruited through social media or flyer distribution, and submitted to online versions of the gelotophobia assessment instrument (GELOPH < 15 >) and the STAI.

**Results:**

Most participants (*N* = 56, 86.15%) had an STAI‐T score indicative of high trait anxiety. The average GELOPH < 15 > score of the sample was 2.69 (0.65) and 39 of the subjects (60%) were considered gelotophobes. There was a strong positive correlation between the GELOPH < 15 > and STAI‐T scores but no correlation between bullying and either the STAI‐T and GELOPH < 15 > scores. However, the great majority of subjects with gelotophobia reported been previously bullied.

**Conclusion:**

In our sample, all gelotophobes had trait anxiety, but only a fraction of anxious subjects had gelotophobia. These preliminary findings expand on previous reports underscoring the high prevalence of mental health problems afflicting higher education students in Brazil.

## Graphical abstract

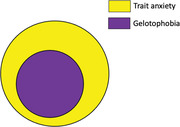

## 1 INTRODUCTION

A recent survey showed that college students are currently experiencing much higher rates of mental health issues than the general population while their institutions are woefully unprepared to handle this crisis (Leonhardt, 2022). According to that report, the most common afflictions are depression and anxiety (Leonhardt, 2022). Even though the survey was performed with US students and during the current COVID‐19 pandemic, a similar high prevalence of mental health complaints was also reported in Brazil earlier: A study with undergraduate students enrolled in Brazilian federal universities revealed a staggering rate of 70% of students with mental health problems, including anxiety, excessive shyness, and depression (FONAPRACE—FÓRUM NACIONAL DE PRÓ‐REITORES DE ASSUNTOS COMUNITÁRIOS E ESTUDANTIS, 2014) while another survey performed with 1650 undergraduate medical students found that 85.6% of them had anxiety (Brenneisen Mayer et al., 2016).

Even without COVID‐19‐related measures such as school closures and social distancing guidelines, the academic routine of college students is beset with anxiogenic situations, such as public speaking and oral assessments (Nash et al., 2016). It is very common for college instructors to meet students who are strongly reluctant to speak in public. Public speaking anxiety (PSA), defined by Bodie (Bodie, 2010) “as a situation specific social anxiety that arises from the real or anticipated enactment of an oral presentation,” is a highly prevalent disorder (Pollard & Henderson, 1988; Stein, 1996), which causes significant distress or impairment in both social and occupational situations (Pull, 2012, Jan). PSA is considered a distinct subtype of social anxiety disorder (SAD) (Blöte et al., 2009), and about half of the adolescents and adults with PSA will eventually develop generalized anxiety (Blöte et al., 2009; Hofmann et al., 1999). However, few studies have evaluated the prevalence of PSA in university students and most have targeted students from northern countries, such as the United States (Dwyer & Davidson, 2012; Ferreira Marinho et al., 2017), the United Kingdom (Russell & Topham, 2012), and Sweden (Tillfors & Furmark, 2007). All those studies reported a high prevalence of PSA among college students. A study in Brazil with a sample of psychology students showed that 63.9% reported fear of speaking in public (Croucher et al., 2015). There is clearly a need for additional studies in this subject, specifically with culturally diverse samples to understand the influence of broad cultural characteristics on PSA. In some countries, such as the United States and England, for instance, it is common for students to receive specific instructions on public speaking or participate in debate groups. This is less common in other countries and, as shown by a Finnish study, English participants scored lower than either Finnish or German participants on communication apprehension, both dyadic and in public (Croucher et al., 2015). In Brazil, there is little to non‐existent curricular emphasis on oral communication skills at all levels of education, from elementary school to the university.

The human brain is wired for social attention and to efficiently process bodily cues from our conspecifics and adjust our behavior appropriately (Klein et al., 2009). For instance, when we are speaking in public, we both consciously and unconsciously search for specific nonverbal signals among the audience to gauge how we are being perceived. Facial expressions play a major part in social interactions and are able to convey a vast repertoire of emotions (Frith, 2009). Studies have shown that the anticipation of a public‐speaking commitment makes subjects oversensitive to manifestations of discomfort or disinterest from the audience and is enough to enhance the processing of angry faces, for instance (e.g. Wieser et al., 2010). However, in the same situation, some individuals with a condition called gelotophobia can interpret positive signs as threatening and be unwilling to speak in front of classmates, for instance (Barabadi et al., 2021).

Gelotophobia was first described as the “pathological fear of being laughed at” (Gelotophobia, 2009) and it shares many characteristics with SAD and avoidant personality disorders (AvPD), such as fear of negative evaluation, shyness, increased attention to social threats, and the presence of cognitive distortions that involve fear and mistrust of other individuals’ intentions (Ruch et al., 2014; Fenigstein & Vanable, 1992). Gelotophobes display a negative association with the accuracy of positively valenced stimuli, such as laughter, and also make more errors than non‐gelotophobes in a gaze discrimination task used to evaluate theory of mind (TOM) (Torres‐Marín et al., 2017). Gelotophobia can be measured by the GELOPH < 15 > questionnaire (Ruch & Proyer, 2008) and its cross‐cultural relevance has been demonstrated elsewhere (Lampert et al., 2010; Proyer et al., 2009). Some questions that remain unanswered about gelotophobia are associated with its developmental underpinnings. For instance, though bullying victimization is positively correlated to the presence of anxiety in children and adolescents (Pontillo et al., 2019), less is known about its associations with gelotophobia (Platt et al., 2009; Proyer et al., 2013; Edwards et al., 2010).

Gelotophobia is a relatively new concept and shares traits with other conditions associated with the introverted neurotic personality type. The State‐Trait Anxiety Inventory (STAI) provides two measures two types of anxiety—state anxiety (STAI‐S) and trait anxiety (STAI‐T) (Spielberger & Gorsuch, 1983). The STAI‐T is a strong indicator for clinical depression and anxiety disorders (Knowles & Olatunji, 2020). Since gelotophobes are often unaware of their condition and may consider themselves as having anxiety instead, in the present study, we recruited a sample of college students who self‐identified as anxious and evaluated the association of their STAI‐T and GELOPH < 15 > scores. Our goal was to have a grasp of (1) the proportion of individuals with gelotophobia among those with trait anxiety, (2) the dimensional characteristics of traits related to trait anxiety, (3) the association of early episodes of bullying with GELOPH < 15 > scores, and (4) contribute to the understanding of the cultural underpinnings of gelotophobia by studying a Brazilian sample.

## 2 MATERIALS AND METHODS

### 2.1 Participants

The volunteers were undergraduate students from the Federal University of Pará (UFPA) recruited either via social networks (Instagram, Facebook, and WhatsApp) or hand‐delivery of recruitment flyers. The inclusion criteria were (I) self‐identification as anxious and (II) being enrolled in the University. Data was gathered via Google Forms. The procedures were approved by the Human Research Ethics Committee of the Federal University of Pará (CAAE: 28646419.4.0000.0018/Number: 3.965.234). All volunteers were informed about the objectives and risks of the research and agreed to participate via free and informed consent.

Initially, 96 volunteers signed up to participate in the study. However, the final sample was composed of 65 subjects (38 females, 21.48 ± 2.54 y.o., 18–28 y.o.), who completed all experimental steps and fully met inclusion criteria.

### 2.2 Experimental procedures

We used the GELOPH < 15 > and the STAI to measure the fear of being laughed at and trait anxiety, respectively. The Portuguese version of the GELOPH < 15 > (Proyer et al., 2009) is based on the self‐reported responses to 15 questions structured in a 4‐point Likert scale (ranging from 1 = “strongly disagree”, 2 = “moderately disagree”, 3 = “moderately agree”, and 4 = “strongly agree”) (Ruch & Proyer, 2008). The individual gelotophobia scores are averaged among the responses to 15 questions, resulting in a total score ranging from 0 to 4. Ruch and Proyer's (2008) study conducted in subclinical samples break down GELOPH < 15 > scores in four levels, namely, without gelotophobia (1.00–1.99), borderline fearful (2–2.49), slight gelotophobia (2.50–2.99), pronounced gelotophobia (3.00–3.49), and extreme gelotophobia (3.50–4.00).

The STAI is composed of two parts, the STAI‐State (STAI‐S) and the STAI‐Trait (STAI‐T) (Spielberger & Gorsuch, 1983), which measure anxiety as a transient and as a lasting and recurrent condition, respectively (Biaggio et al., 1977). In this study, only trait anxiety analysis (STAI‐T) will be used. Each questionnaire consists of 20 self‐report questions based on a 4‐point Likert scale. The results were summed, resulting in a score ranging from 20 to 80 points (Spielberger & Gorsuch, 1983).

Participants were also asked about their personal experience with bullying through four questions: “Have you ever been bullied?”, “If so, when?”, “If so, where?”, “What type of bullying?” The candidate could choose more than one answer from the following (depending on the answer to the first question): “yes,” “no, I don't know”; “childhood, adolescence, currently”; “at home, in my neighborhood, at school, at college; work, in social situations (parties/events)”; “psychological (offenses, threats, exclusion, body perception, racism, sexism, LGBTQIA+‐phobia, etc.), physical (aggressions), sexual (harassment and/or abuse), others.”

### 2.3 Statistical analysis

The statistical analyses were performed with the Statistical Package for Social Sciences (IBM SPSS®) software. The responses to the questionnaires and sociodemographic variables were analyzed with independent *t*‐test, Mann–Whitney *U* test, and Fisher's exact test to find possible statistical associations between the answers to the questionnaires and the characteristics of the participants (sex, age, and occupation).

A simple linear regression was performed between individual STAI‐T and GELOPH < 15 > scores. A Spearman correlation compared the association between the two test scores (GELOPH and STAI) variables with the occurrence of Bullying. The level of significance was set at 0.05.

## 3 RESULTS

### 3.1 Sample

The final experimental sample consisted of 65 participants (38 females), aged between 18 and 28 years (21.48 ± 2.54 y.o.) (Table 1). The subjects were enrolled in the following majors: Engineering, Accounting, Nutrition, Dentistry, Literature, Law, Nursing, Psychology, Philosophy, Administration, Pedagogy, and Social Communication. Among them, 43(66.15%) declared only studying and 22(33.85%) declared both studying and working (Table 1).

**TABLE 1:** Sociodemographic characteristics of participants

		Female	Male	*p* value
**Age [Mean (SD)]**		21.21 (2.22)	21.85 (2.93)	0.319
**Occupation [*N* (%)]**				
	Study	23 (53.49)	20 (46.51)	0.254
	Study and Work	15 (68.18)	7 (31.82)	

### 3.2 Individual questionnaires

Shapiro–Wilk tests showed that both GELOPH < 15 > (*W* = 0.97, *p* > 0.05) and STAI‐T scores (*W* = 0.96, *p* = 0.05) have a normal distribution. Thus, we used mean and standard deviation to characterize the variables.

The average STAI‐T score of the sample is 56.23(10.31) and most participants (N = 56, 86.15%) have a STAI‐T score above the Brazilian average of 46 (Biaggio et al., 1977) (Table 2). There is no statistical difference between the means of STAI‐T in males (M = 57.33, SD = 9.54) and females (M = 55.45, SD = 10.8) (t(63) = 0.724, *p* > 0.05) and between those who are only students (M = 55.81, SD = 9.53) and those who also work (M = 57.05, SD = 11.9) (t(63) = –0.453, *p* > 0.05) (Table 3). No association was found between the STAI‐T scores and age ($\chi^{2}\;\left(279\right)=\;258,\;p>\;0.05$) using Fisher's exact test.

**TABLE 2:** Descriptive statistics for gelotophobia and anxiety scores

	Mean	SD	Skewness	Kurtosis
**STAI‐T**	56.23	10.31	–0.71	–0.73
**GELOPH < 15 >**	2.698	0.64	–0.27	–0.42

**TABLE 3:** Questionnaire results by sex

				Female	Male
GELOPH < 15 >				*N* (%)	
			Without gelotophobia (1–1.99)	6 (9.23)	1 (1.54)
			Borderline fearful (2–2.49)	9 (13.85)	10 (15.38)
			Mild gelotophobia (2.5–2.99)	4 (6.15)	7 (10.77)
			Pronounced gelotophobia (3–3.49)	15 (23.08)	8 (12.31)
			Extreme gelotophobia (3.5–4)	4 (6.15)	1 (1.54)
**Bullying**				**N (%)**	
	No			7 (10.77)	1 (1.54)
	I don't know			3 (4.61)	5 (7.69)
	Yes			28 (43.08)	21 (32.31)
		Developmental Phase	Childhood	19	18
			Adolescence	17	16
			Now	6	2
		Place	Home	10	7
			Neighborhood	7	7
			School	28	21
			College	5	2
			Work	2	2
			Social situations (parties/events)	5	5
		Typology	Psychological	27	20
			Physical	6	6
			Sexual	6	2
			Others	2	1
**STAI‐T**				**Mean (SD)**	
				56.23 (10.88)	57.33 (9.53)

The average GELOPH < 15 > score of our sample is 2.69(.65) (Table 2). Out of the 65 participants, 39(60%) have gelotophobia, with 11 having mild gelotophobia (16.92%), 23 pronounced gelotophobia (35.38%), and 5 extreme gelotophobia (7.7%) (Table 3). There is no statistical difference between the means of GELOPH < 15 > in males (M = 2.71, SD = .54) and females (M = 2.69, SD = .72), t(62.8)=0.111,p>0.05, and between those who are only students (M = 2.69, SD = 0.62) and those who also work (M = 2.72, SD = 0.71), t(37.8)=−.193,p>0.05 (Table 4). There is no association between GELOPH < 15 > scores and age $\left(\chi^{2}\;\left(279\right)=\;278,\;p>\;0.05\right)$ using Fisher's exact test (Table 4).

**TABLE 4:** Effect of age, sex (male and female), and occupation (study or study and work) on response to the questionnaires

	p‐value		
	Sex	Age	Occupation
**STAI‐T**	0.476^1^	0.802^2^	0.437^1^
**GELOPH < 15 >**	0.911^1^	0.495^2^	0.848^1^
**Bullying**	0.485^2^	0.436^2^	0.671^2^

^1^Mann–Whitney *U* test;

^2^Independent *t*‐test;

^3^Fisher's Exact Test

Regarding bullying, 49 participants indicated they had been bullied (75.38%), while 8 answered “No” (12.31%), and 8 “I don't know” (12.31%) (Table 3). Fisher's exact test found no association in the experience of bullying between males and females $(\chi^{2}\;\left(2\right)=\;1.676,\;p>\;0.05$), nor between types of occupation ($\chi^{2}\;\left(2\right)=\;1.277,\;p>\;0.05$), nor between age ($\chi^{2}\;\left(18\right)=\;16.56,\;p>\;0.05$) (Table 4). Most subjects suffered bullying at school (49/49) during their childhood (37/49) and associated with psychological aggression (offense, threats, exclusion, body perception, racism, sexism, LGBTQIA+‐phobia, etc.) (47/49) (Table 3).

### 3.3 Paired analyses

#### 3.3.1 STAI and GELOPH < 15 >

**FIGURE 1:**
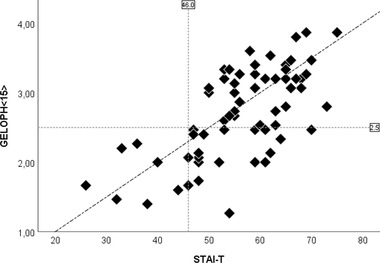
Scatter plot depicting the association between STAI and GELOPH < 15 > scores. The vertical line represents the Brazilian STAI population average (46); the horizontal line represents the cut‐off point of GELOPH < 15 > (2.5).

Since both distributions do not have outliers, we expect these parameters to not affect the size of the correlations between STAI‐T and GELOPH < 15 > scores (Proyer et al., 2009). There is a strong positive correlation between GELOPH < 15 > and STAI‐T scores (r(65)=0.667,p<0.001). A simple linear regression was calculated to predict the GELOPH < 15 > scores based on STAI‐T scores. A significant regression equation was found ($F\;\left(1,\;63\right)=\;50.4,\;p<0.001,\;\;R^{2}=\;0.445$). Participants’ GELOPH < 15 > scores increased 0.042 to each STAI‐T score increase. All subjects with gelotophobia were also above the Brazilian population average for anxiety (Ruch & Proyer, 2008) (see Figure 1).

#### 3.3.2 STAI and bullying

**FIGURE 2:**
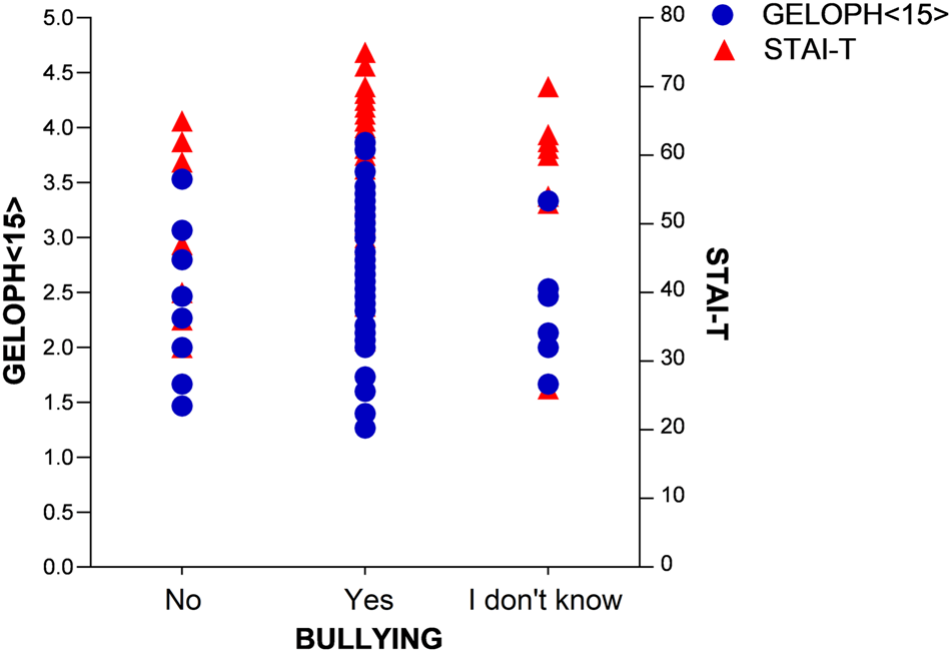
Relationship between bullying experience, STAI‐T, and GELOPH < 15 > scores.

There was no significant correlation between STAI‐T scores and bullying (ρ(65)=0.199,p>0.05). Figure 2 shows the distribution of individual STAI‐T and GELOPH < 15 > scores according to bullying experience. However, of the 56 participants with STAI‐T scores above 46 (Brazilian average, see (Ruch & Proyer, 2008)), 45(80.36%) stated that they had been bullied while only 7(12.5%) were unable to confirm it and 4(7.14%) claimed not to have been bullied.

#### 3.3.3 GELOPH < 15 > and bullying

There was no significant correlation between GELOPH < 15 > scores and Bullying (ρ(65)=−0.014,p>0.05). Figure 2 shows the distribution of GELOPH < 15 > scores and bullying responses. Of the 49 participants who reported been bullied, 4(8.16%) had “extreme gelotophobia,” 21(42.86%) had “pronounced gelotophobia,” 8(16.33%) had “mild gelotophobia,” 12(24.49%) were “borderline fearful,” and 4(8.16%) were “without gelotophobia.”

## 4 DISCUSSION

The majority (60%) of subjects in our sample were gelotophobes, with an average GELOPH < 15 > score of 2.69(0.08), higher than the average score (mean = 1.67, SD = 0.52) reported in a previous study (Proyer et al., 2009) including a Brazilian sample (*N* = 211) (mean age = 37.2, SD = 13.9). Different from that earlier study, however, we targeted college students who self‐identified as anxious and found a strong positive correlation between GELOPH < 15 > and STAI‐T scores. This finding reinforces the robust relationship patterns between anxiety and the fear of being laughed at reported in previous studies (Havranek et al., 2017; Carretero‐Dios et al., 2010; Torres‐Marín et al., 2021). Even though GELOPH < 15 > and STAI‐T scores were strongly correlated in our study, there was a large proportion of anxious subjects who were not gelotophobes (30.35%) (Figure 1). This result supports the findings from previous studies (Gelotophobia, 2009; Edwards et al., 2010; Carretero‐Dios et al., 2010) proposing the fear of being laughed at cannot fully be accounted for by anxiety measures and thus should be considered different constructs.

Gelotophobia seems to be associated with traumatic experiences of being ridiculed during childhood and adolescence, making gelotophobes deeply afraid of being exposed to shame‐inducing situations (Platt et al., 2009). Previous studies have reported an association between bullying/teasing and gelotophobia (Platt et al., 2009; Edwards et al., 2010; Führ, 2010; Platt, 2021; Proyer et al., 2011, 2013). In our study, though we found no significant correlation between gelotophobia and bullying in college students, the great majority of subjects with gelotophobia reported been previously bullied.

The present study is the first focusing specifically on gelotophobia in undergraduate students in Brazil. Previous studies had shown a large rate of anxiety complaints by those students (Fonaprace—fórum nacional de pró‐reitores de assuntos comunitários e estudantis. 2014; Brenneisen Mayer et al., 2016). Given that gelotophobia is a relatively new concept and shares traits with other conditions associated with the introverted neurotic personality type, such as SAD, and our own results, we expect the fear of being laughed at could be highly prevalent in higher education throughout Brazil.

The cross‐cultural underpinnings of gelotophobia have been the focus of relatively few studies (Lampert et al., 2010; Proyer et al., 2009). The study by Proyer et al. (2009) evaluated 93 samples from 73 countries and showed that gelotophobia is found in many cultures around the world. However, that pioneer study made no distinction of the internal variations in the ethnic and racial compositions of some large countries, such as the United States and Brazil. A later study by Lampert et al. (2010) evaluated the difference between European and Asian American GELOPH < 15 > respondents in a US sample. Their results showed that subtle cultural differences play an important role in the development and manifestation of gelotophobia (Lampert et al., 2010). Thus, our present results represent a cross‐section of a sample with rather similar demographic characteristics, but which do not represent the entire Brazilian population, even when only college students are considered. Like the United States, Brazil has a very diverse ethnic and racial composition with great inter‐regional variation. We plan a future study with a larger sample, more representative of the actual Brazilian demographic composition.

One limitation of our study was the relatively small sample size. The study had a high attrition rate (32.29%), mostly due to participants abstaining from responding to the complete questionnaire set, including both the GELOPH < 15 > and the STAI. However, our aim was to evaluate the occurrence of gelotophobia in college students self‐identifying as anxious. The study was performed online due to restrictions associated with COVID‐19 and the dropout rates we encountered (32.29%) conform to expectations of online surveys (Lumsden et al., 2017), with the caveat that participation in our study was unpaid as required by Brazilian legislation.

## 5 CONCLUSION

Altogether, our results show a large proportion of gelotophobia in a sample of Brazilian undergraduate college students self‐identifying with anxiety. In that sample, all gelotophobes had trait anxiety, but only a fraction of anxious subjects had gelotophobia. These results highlight the need to understand the harmful effects of gelotophobia in higher education, such as the negative consequences on academic performance associated with PSA. Finally, we hope the present work also contributes to the understanding of the cultural underpinnings of gelotophobia.

## AUTHOR CONTRIBUTIONS

Tainá S. Rêgo and Antonio Pereira designed the study and wrote the manuscript; Juan‐Pablo Aristizabal provided the STAI; Tainá S. Rêgo and Diógenes E. S. Pires collected the data; Tainá S. Rêgo, Diógenes E. S. Pires, Eline M. Melo, and Antonio Pereira analyzed the data; all authors analyzed and commented on the manuscript.

## CONFLICT OF INTEREST

The authors declare that there are no conflict of interests.

### PEER REVIEW

The peer review history for this article is available at https://publons.com/publon/10.1002/brb3.2757.

## Data Availability

The ethics committee did not grant permission to share study data with third parties or to upload data in anonymized form.
